# 7-[(5,5-Dimethyl-2-oxido-1,3,2-dioxaphosphinan-2-yl)­oxy]-4-methyl-2*H*-chromen-2-one

**DOI:** 10.1107/S1600536812023513

**Published:** 2012-05-31

**Authors:** Yan-Ru Zhao, Xu-Feng Hou, Zhi-Hong Xu

**Affiliations:** a College of Chemistry and Chemical Engineering, Xuchang University, Xuchang, Henan Province 461000, People’s Republic of China

## Abstract

The title compound, C_15_H_17_O_6_P, was obtained from a reaction of 4-methyl-7-hy­droxy­coumarin and 2-chloro-5,5-dimethyl-1,3,2-dioxaphosphinane 2-oxide. There are two mol­ecules in the asymmetric unit in which the benzopyran ring system is almost planar [r.m.s. deviation for each molecule = 0.003 Å]. In the crystal, C—H⋯O hydrogen bonds and π–π stacking inter­actions [with centroid–centroid distances of 3.743 (3) and 3.727 (3) Å] link the two mol­ecules. The dioxaphospho­rinane ring adopts a chair conformation in both asymmetric molecules.

## Related literature

For the application of 4-methyl-7-hy­droxy­coumarin and 2-oxido-1,3,2-dioxaphosphinan derivatives, see: Babu *et al.*(2008 ▶); Li *et al.* (2002 ▶, 2006 ▶); Raghu & Reddy (1996 ▶); Sierosławski *et al.* (2006 ▶); Zhou *et al.* (2006 ▶).
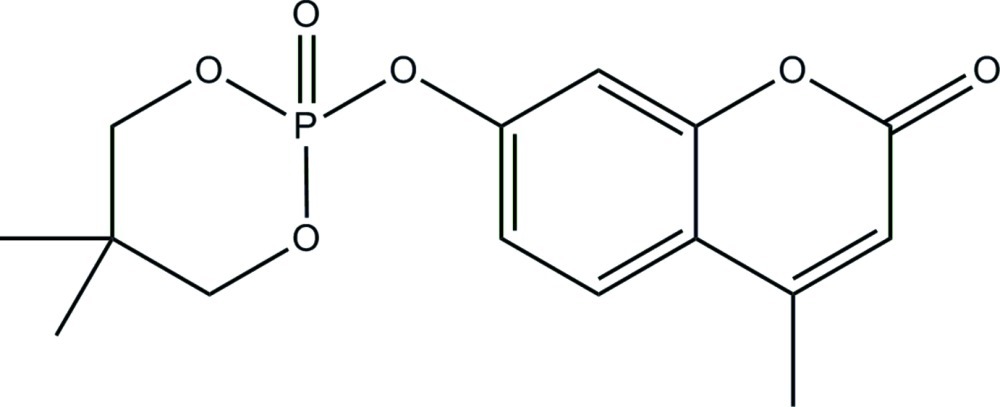


## Experimental

### 

#### Crystal data

C_15_H_17_O_6_P
*M* *_r_* = 324.26
Monoclinic, 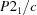

*a* = 7.309 (4) Å
*b* = 17.010 (9) Å
*c* = 25.507 (13) Å
β = 102.596 (17)°
*V* = 3095 (3) Å^3^
*Z* = 8
Mo *K*α radiation
μ = 0.20 mm^−1^
*T* = 293 K
0.22 × 0.17 × 0.15 mm

#### Data collection

Bruker APEXII CCD diffractometer
Absorption correction: multi-scan (*SADABS*; Bruker, 2007 ▶) *T*_min_ = 0.957, *T*_max_ = 0.970
16892 measured reflections
6065 independent reflections
2926 reflections with *I* > 2σ(*I*)
*R*_int_ = 0.077

#### Refinement

*R*[*F* ^2^ > 2σ(*F* ^2^)] = 0.052
*wR*(*F* ^2^) = 0.134
*S* = 1.00
6065 reflections
398 parameters
H-atom parameters constrained
Δρ_max_ = 0.24 e Å^−3^
Δρ_min_ = −0.23 e Å^−3^

### 

Data collection: *APEX2* (Bruker, 2007 ▶); cell refinement: *SAINT* (Bruker, 2007 ▶); data reduction: *SAINT*; program(s) used to solve structure: *SHELXS97* (Sheldrick, 2008 ▶); program(s) used to refine structure: *SHELXL97* (Sheldrick, 2008 ▶); molecular graphics: *SHELXTL* (Sheldrick, 2008 ▶); software used to prepare material for publication: *SHELXTL*.

## Supplementary Material

Crystal structure: contains datablock(s) global, I. DOI: 10.1107/S1600536812023513/ez2291sup1.cif

Structure factors: contains datablock(s) I. DOI: 10.1107/S1600536812023513/ez2291Isup2.hkl

Supplementary material file. DOI: 10.1107/S1600536812023513/ez2291Isup3.cml

Additional supplementary materials:  crystallographic information; 3D view; checkCIF report

## Figures and Tables

**Table 1:** Hydrogen-bond geometry (Å, °)

*D*—H⋯*A*	*D*—H	H⋯*A*	*D*⋯*A*	*D*—H⋯*A*
C8—H8*A*⋯O2^i^	0.93	2.36	3.223 (5)	155
C13—H13*B*⋯O11^ii^	0.97	2.48	3.250 (5)	136

Symmetry codes: (i) 
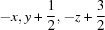
; (ii) 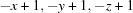.

**Fig. 1.**
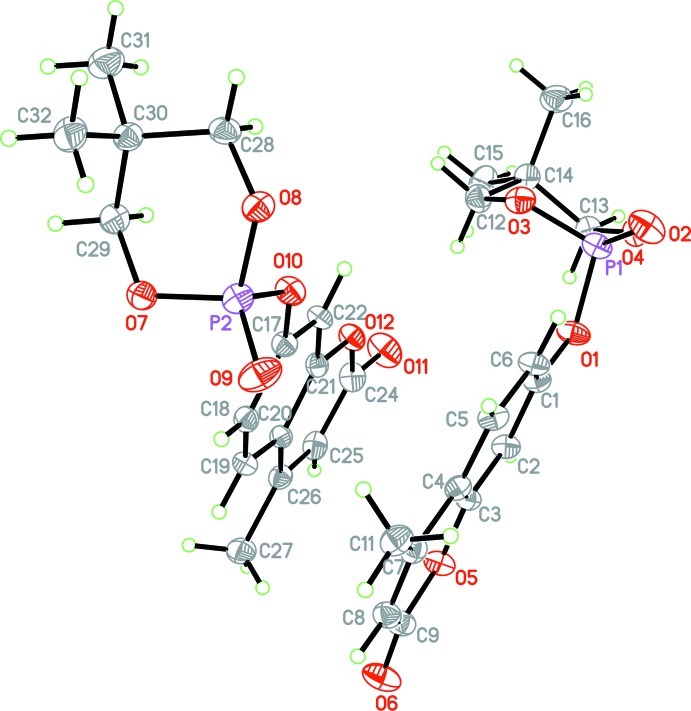
The asymmetric unit of the title compound, (I), with displacement ellipsoids drawn at the 30% probability level.

**Fig. 2.**
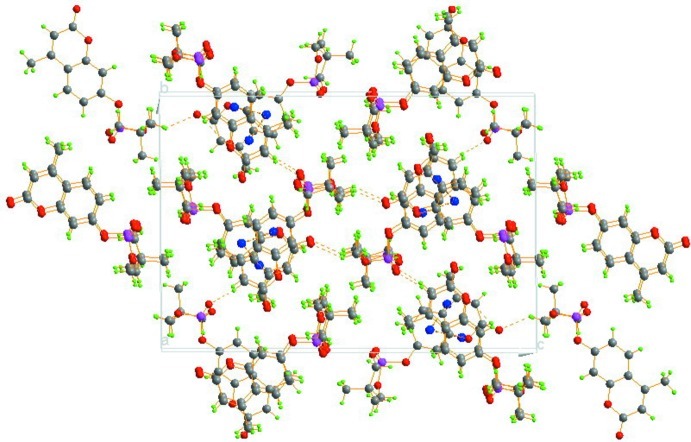
The partial packing diagram of the title compound, hydrogen bonds and π-π interactions are indicated by dashed lines, with centroids indicated in blue.

## supplementary crystallographic information

### Comment

2-oxido-1,3,2-dioxaphosphinan and its derivatives exhibit high flame-retardance (Li *et al.*, 2002; Li *et al.*, 2006) as well as biological and pharmaceutical activity (Babu *et al.*, 2008). Coumarin and its derivatives have a wide range of biological activities, and for years have received significant attention regarding natural and synthetic sources (Sierosławski *et al.*, 2006; Zhou *et al.*, 2006). Few crystal sructures containing both of these groups have been characterized. We report here the crystal structure of a new 2-chloro-1,3,2-dioxaphosphinane derivative containing the 7-oxy-4-methyl-2*H*-chromen-2-one group.

In the title compound, C_15_H_17_O_6_P, bond distances and angles in (I) are as expected, and the dioxaphosphinan ring adopts a chair conformation (Fig. 1). π-π stacking interactions and C—H···O hydrogen bonds link the molecules. (Table 1, Fig. 2)

### Experimental

The title compound was prepared according to the procedure of Raghu & Reddy (1996). The 4-methyl-7-hydroxycoumarin (0.95 g, 6.2 mmol), dry dichloromethane (10 ml) and triethylamine (0.61 g, 6 mmol) were placed in a 100 ml three-necked flask and a solution of 2-chloro-5,5-dimethyl- [1,3,2]dioxaphosphinane 2-oxide (1.12 g, 6.1 mmol) in dry dichloromethane (5 ml) was added dropwise over a period of 1 h at room temperature (298 K). The reaction temperature was raised to 308 K and stirring was continued for 8 h. The solvent was removed under reduced pressure and the residual mixture was washed with anhydrous ether (25 ml), dried and recrystallized from ethanol to give compound (I). Suitable crystals were obtained from a anhydrous methanol at room temperature (m.p. 440 K).

### Refinement

All H atoms were placed in calculated positions, with C—H = 0.98 Å or 0.99 Å, and included in the final cycles of refinement using a riding model, with *U*_iso_(H) = 1.2Ueq(C).

### Crystal data

**Table:**

C_15_H_17_O_6_P	*F*(000) = 1360
*M**_r_* = 324.26	*D*_x_ = 1.392 Mg m^−^^3^
Monoclinic, *P*2_1_/*c*	Mo *K*α radiation, λ = 0.71073 Å
*a* = 7.309 (4) Å	Cell parameters from 1226 reflections
*b* = 17.010 (9) Å	θ = 2.4–20.1°
*c* = 25.507 (13) Å	µ = 0.20 mm^−^^1^
β = 102.596 (17)°	*T* = 293 K
*V* = 3095 (3) Å^3^	Block, colorless
*Z* = 8	0.22 × 0.17 × 0.15 mm

### Data collection

**Table:**

Bruker APEXII CCD diffractometer	6065 independent reflections
Radiation source: fine-focus sealed tube	2926 reflections with *I* > 2σ(*I*)
Graphite monochromator	*R*_int_ = 0.077
φ and ω scans	θ_max_ = 26.0°, θ_min_ = 1.5°
Absorption correction: multi-scan (*SADABS*; Bruker, 2007)	*h* = −9→7
*T*_min_ = 0.957, *T*_max_ = 0.970	*k* = −20→20
16892 measured reflections	*l* = −29→31

### Refinement

**Table:**

Refinement on *F*^2^	Secondary atom site location: difference Fourier map
Least-squares matrix: full	Hydrogen site location: inferred from neighbouring sites
*R*[*F*^2^ > 2σ(*F*^2^)] = 0.052	H-atom parameters constrained
*wR*(*F*^2^) = 0.134	*w* = 1/[σ^2^(*F*_o_^2^) + (0.040*P*)^2^] where *P* = (*F*_o_^2^ + 2*F*_c_^2^)/3
*S* = 1.00	(Δ/σ)_max_ = 0.001
6065 reflections	Δρ_max_ = 0.24 e Å^−^^3^
398 parameters	Δρ_min_ = −0.23 e Å^−^^3^
0 restraints	Extinction correction: *SHELXL97* (Sheldrick, 2008), Fc^*^=kFc[1+0.001xFc^2^λ^3^/sin(2θ)]^-1/4^
Primary atom site location: structure-invariant direct methods	Extinction coefficient: 0.0028 (4)

### Special details

**Table:**

Geometry. All e.s.d.'s (except the e.s.d. in the dihedral angle between two l.s. planes) are estimated using the full covariance matrix. The cell e.s.d.'s are taken into account individually in the estimation of e.s.d.'s in distances, angles and torsion angles; correlations between e.s.d.'s in cell parameters are only used when they are defined by crystal symmetry. An approximate (isotropic) treatment of cell e.s.d.'s is used for estimating e.s.d.'s involving l.s. planes.
Refinement. Refinement of *F*^2^ against ALL reflections. The weighted *R*-factor *wR* and goodness of fit *S* are based on *F*^2^, conventional *R*-factors *R* are based on *F*, with *F* set to zero for negative *F*^2^. The threshold expression of *F*^2^ > σ(*F*^2^) is used only for calculating *R*-factors(gt) *etc*. and is not relevant to the choice of reflections for refinement. *R*-factors based on *F*^2^ are statistically about twice as large as those based on *F*, and *R*- factors based on ALL data will be even larger.

### Fractional atomic coordinates and isotropic or equivalent isotropic displacement parameters (Å^2^)

**Table:**

	*x*	*y*	*z*	*U*_iso_*/*U*_eq_	
P1	0.16499 (15)	0.36220 (5)	0.60542 (4)	0.0508 (3)	
P2	0.55845 (14)	0.45430 (5)	0.91243 (4)	0.0506 (3)	
C1	0.1215 (5)	0.49770 (19)	0.65432 (12)	0.0456 (9)	
C2	0.1394 (4)	0.57797 (18)	0.65058 (12)	0.0460 (9)	
H2A	0.1587	0.6008	0.6191	0.055*	
C3	0.1282 (4)	0.62402 (18)	0.69453 (13)	0.0435 (8)	
C4	0.1021 (4)	0.59221 (19)	0.74286 (12)	0.0411 (8)	
C5	0.0811 (5)	0.51023 (19)	0.74467 (13)	0.0485 (9)	
H5A	0.0615	0.4871	0.7760	0.058*	
C6	0.0887 (5)	0.46305 (18)	0.70101 (13)	0.0490 (9)	
H6A	0.0722	0.4090	0.7028	0.059*	
C7	0.0929 (4)	0.6455 (2)	0.78687 (12)	0.0452 (8)	
C8	0.1078 (5)	0.7232 (2)	0.77870 (13)	0.0515 (9)	
H8A	0.1027	0.7573	0.8069	0.062*	
C9	0.1312 (5)	0.7563 (2)	0.72885 (15)	0.0551 (10)	
C11	0.0707 (5)	0.6132 (2)	0.84025 (13)	0.0574 (10)	
H11A	0.0663	0.6558	0.8646	0.086*	
H11B	0.1752	0.5797	0.8548	0.086*	
H11C	−0.0433	0.5834	0.8353	0.086*	
C12	0.5251 (5)	0.3759 (2)	0.60956 (14)	0.0607 (10)	
H12A	0.6443	0.3559	0.6295	0.073*	
H12B	0.5298	0.4329	0.6113	0.073*	
C13	0.3071 (5)	0.3804 (2)	0.52109 (13)	0.0526 (9)	
H13A	0.3079	0.4374	0.5219	0.063*	
H13B	0.2864	0.3640	0.4838	0.063*	
C14	0.4956 (5)	0.3501 (2)	0.55123 (13)	0.0530 (9)	
C15	0.6481 (6)	0.3882 (2)	0.52679 (17)	0.0873 (14)	
H15A	0.6404	0.4444	0.5294	0.131*	
H15B	0.6302	0.3733	0.4897	0.131*	
H15C	0.7692	0.3708	0.5460	0.131*	
C16	0.5025 (6)	0.2598 (2)	0.54750 (16)	0.0778 (12)	
H16A	0.4061	0.2374	0.5630	0.117*	
H16B	0.6227	0.2414	0.5666	0.117*	
H16C	0.4831	0.2444	0.5105	0.117*	
C17	0.5687 (4)	0.51941 (18)	0.81753 (12)	0.0419 (8)	
C18	0.5652 (4)	0.59612 (18)	0.83640 (12)	0.0437 (8)	
H18A	0.5496	0.6059	0.8710	0.052*	
C19	0.5856 (4)	0.65767 (18)	0.80243 (13)	0.0446 (8)	
H19A	0.5867	0.7090	0.8150	0.054*	
C20	0.6046 (4)	0.64455 (17)	0.74978 (12)	0.0386 (8)	
C21	0.6056 (4)	0.56604 (18)	0.73303 (12)	0.0401 (8)	
C22	0.5888 (5)	0.50344 (17)	0.76621 (12)	0.0421 (8)	
H22A	0.5910	0.4519	0.7542	0.051*	
C24	0.6378 (5)	0.6043 (2)	0.64460 (15)	0.0577 (10)	
C25	0.6431 (5)	0.6848 (2)	0.66261 (14)	0.0554 (10)	
H25A	0.6580	0.7241	0.6385	0.066*	
C26	0.6277 (4)	0.70635 (19)	0.71221 (13)	0.0452 (9)	
C27	0.6342 (5)	0.79147 (18)	0.72920 (14)	0.0632 (11)	
H27A	0.6497	0.8242	0.6998	0.095*	
H27B	0.7377	0.7994	0.7591	0.095*	
H27C	0.5194	0.8050	0.7395	0.095*	
C28	0.7311 (5)	0.32002 (18)	0.91740 (14)	0.0540 (10)	
H28A	0.7330	0.3180	0.8795	0.065*	
H28B	0.7229	0.2665	0.9298	0.065*	
C29	0.9198 (5)	0.44192 (19)	0.92909 (14)	0.0537 (10)	
H29A	1.0346	0.4666	0.9483	0.064*	
H29B	0.9210	0.4421	0.8911	0.064*	
C30	0.9136 (5)	0.35703 (19)	0.94827 (13)	0.0499 (9)	
C31	1.0766 (5)	0.3125 (2)	0.93284 (17)	0.0774 (12)	
H31A	1.0650	0.3153	0.8947	0.116*	
H31B	1.0736	0.2584	0.9435	0.116*	
H31C	1.1933	0.3357	0.9507	0.116*	
C32	0.9275 (5)	0.3540 (2)	1.00886 (13)	0.0672 (11)	
H32A	0.8241	0.3821	1.0175	0.101*	
H32B	1.0432	0.3777	1.0271	0.101*	
H32C	0.9240	0.3002	1.0201	0.101*	
O1	0.1328 (3)	0.45455 (12)	0.60837 (8)	0.0557 (7)	
O2	0.0340 (4)	0.31360 (13)	0.62644 (10)	0.0716 (8)	
O3	0.3740 (3)	0.34751 (13)	0.63452 (8)	0.0571 (7)	
O4	0.1532 (3)	0.35143 (12)	0.54432 (8)	0.0491 (6)	
O5	0.1455 (3)	0.70424 (12)	0.68781 (9)	0.0529 (6)	
O6	0.1404 (4)	0.82590 (15)	0.71832 (11)	0.0785 (9)	
O7	0.7588 (3)	0.48739 (12)	0.93772 (8)	0.0511 (6)	
O8	0.5655 (3)	0.36372 (12)	0.92416 (9)	0.0525 (6)	
O9	0.4070 (4)	0.49634 (15)	0.92830 (10)	0.0756 (8)	
O10	0.5482 (3)	0.45393 (12)	0.84883 (8)	0.0509 (6)	
O11	0.6436 (4)	0.58166 (15)	0.59995 (10)	0.0844 (9)	
O12	0.6214 (3)	0.54691 (12)	0.68164 (8)	0.0495 (6)	

### Atomic displacement parameters (Å^2^)

**Table:**

	*U*^11^	*U*^22^	*U*^33^	*U*^12^	*U*^13^	*U*^23^
P1	0.0694 (7)	0.0429 (6)	0.0452 (6)	−0.0023 (5)	0.0238 (5)	−0.0005 (4)
P2	0.0541 (7)	0.0545 (6)	0.0454 (6)	0.0025 (5)	0.0157 (5)	0.0086 (5)
C1	0.050 (2)	0.047 (2)	0.042 (2)	0.0073 (16)	0.0148 (19)	0.0021 (17)
C2	0.057 (2)	0.042 (2)	0.042 (2)	0.0047 (16)	0.0192 (19)	0.0072 (16)
C3	0.043 (2)	0.035 (2)	0.054 (2)	0.0004 (15)	0.0168 (18)	−0.0011 (16)
C4	0.038 (2)	0.047 (2)	0.041 (2)	0.0041 (15)	0.0147 (17)	0.0048 (16)
C5	0.052 (2)	0.048 (2)	0.049 (2)	0.0069 (17)	0.0200 (19)	0.0110 (17)
C6	0.067 (3)	0.0349 (19)	0.050 (2)	0.0038 (17)	0.021 (2)	0.0023 (16)
C7	0.037 (2)	0.057 (2)	0.044 (2)	0.0050 (16)	0.0134 (17)	−0.0032 (18)
C8	0.052 (2)	0.052 (2)	0.054 (2)	0.0008 (17)	0.018 (2)	−0.0106 (18)
C9	0.055 (3)	0.047 (2)	0.069 (3)	−0.0022 (19)	0.026 (2)	−0.006 (2)
C11	0.055 (2)	0.070 (3)	0.050 (2)	0.0107 (19)	0.018 (2)	0.0032 (19)
C12	0.063 (3)	0.060 (2)	0.056 (2)	−0.004 (2)	0.006 (2)	−0.0031 (19)
C13	0.058 (3)	0.059 (2)	0.045 (2)	−0.0046 (18)	0.020 (2)	0.0057 (17)
C14	0.054 (2)	0.059 (2)	0.049 (2)	−0.0004 (19)	0.016 (2)	−0.0016 (18)
C15	0.058 (3)	0.118 (4)	0.093 (3)	−0.013 (2)	0.030 (3)	0.012 (3)
C16	0.083 (3)	0.072 (3)	0.080 (3)	0.017 (2)	0.021 (3)	−0.014 (2)
C17	0.046 (2)	0.040 (2)	0.0402 (19)	−0.0004 (15)	0.0101 (17)	0.0040 (16)
C18	0.045 (2)	0.051 (2)	0.0354 (19)	0.0023 (16)	0.0078 (17)	−0.0046 (16)
C19	0.048 (2)	0.037 (2)	0.048 (2)	0.0004 (15)	0.0088 (18)	−0.0055 (16)
C20	0.038 (2)	0.0365 (19)	0.042 (2)	−0.0024 (15)	0.0087 (16)	0.0005 (16)
C21	0.042 (2)	0.044 (2)	0.0341 (19)	−0.0020 (15)	0.0084 (17)	−0.0028 (15)
C22	0.052 (2)	0.0331 (18)	0.042 (2)	−0.0041 (15)	0.0119 (18)	−0.0045 (15)
C24	0.060 (3)	0.067 (3)	0.048 (2)	−0.008 (2)	0.016 (2)	0.005 (2)
C25	0.054 (3)	0.060 (3)	0.050 (2)	−0.0088 (18)	0.005 (2)	0.0160 (19)
C26	0.043 (2)	0.045 (2)	0.047 (2)	0.0002 (16)	0.0068 (18)	0.0094 (17)
C27	0.068 (3)	0.042 (2)	0.077 (3)	0.0008 (18)	0.011 (2)	0.0127 (19)
C28	0.066 (3)	0.039 (2)	0.057 (2)	0.0014 (18)	0.013 (2)	0.0092 (17)
C29	0.051 (2)	0.052 (2)	0.060 (2)	−0.0040 (18)	0.017 (2)	0.0068 (18)
C30	0.049 (2)	0.047 (2)	0.053 (2)	−0.0001 (17)	0.0114 (19)	0.0069 (18)
C31	0.064 (3)	0.074 (3)	0.094 (3)	0.016 (2)	0.018 (3)	0.001 (2)
C32	0.077 (3)	0.064 (3)	0.055 (2)	−0.003 (2)	0.004 (2)	0.016 (2)
O1	0.0875 (19)	0.0404 (14)	0.0452 (14)	0.0077 (12)	0.0273 (14)	−0.0003 (11)
O2	0.097 (2)	0.0596 (16)	0.0728 (18)	−0.0196 (14)	0.0503 (17)	−0.0021 (13)
O3	0.0720 (19)	0.0574 (15)	0.0420 (14)	0.0101 (13)	0.0128 (14)	0.0058 (12)
O4	0.0529 (15)	0.0597 (15)	0.0375 (13)	−0.0065 (12)	0.0159 (12)	−0.0056 (11)
O5	0.0698 (18)	0.0399 (14)	0.0554 (14)	0.0002 (12)	0.0278 (14)	−0.0014 (11)
O6	0.111 (2)	0.0421 (16)	0.093 (2)	−0.0051 (14)	0.045 (2)	−0.0051 (14)
O7	0.0574 (16)	0.0471 (14)	0.0484 (14)	0.0004 (12)	0.0110 (13)	−0.0003 (11)
O8	0.0511 (15)	0.0495 (14)	0.0585 (15)	−0.0022 (12)	0.0157 (13)	0.0161 (12)
O9	0.072 (2)	0.093 (2)	0.0701 (18)	0.0306 (15)	0.0334 (16)	0.0169 (15)
O10	0.0664 (17)	0.0441 (14)	0.0429 (13)	−0.0051 (11)	0.0135 (13)	0.0063 (11)
O11	0.129 (3)	0.086 (2)	0.0438 (16)	−0.0146 (18)	0.0305 (18)	−0.0039 (15)
O12	0.0644 (17)	0.0501 (14)	0.0354 (13)	−0.0054 (12)	0.0141 (12)	−0.0035 (11)

### Geometric parameters (Å, º)

**Table:**

P1—O2	1.453 (2)	C15—H15C	0.9600
P1—O4	1.553 (2)	C16—H16A	0.9600
P1—O3	1.566 (3)	C16—H16B	0.9600
P1—O1	1.593 (2)	C16—H16C	0.9600
P2—O9	1.447 (2)	C17—C22	1.376 (4)
P2—O8	1.568 (2)	C17—C18	1.393 (4)
P2—O7	1.570 (3)	C17—O10	1.397 (3)
P2—O10	1.607 (2)	C18—C19	1.388 (4)
C1—C2	1.377 (4)	C18—H18A	0.9300
C1—C6	1.396 (4)	C19—C20	1.398 (4)
C1—O1	1.400 (3)	C19—H19A	0.9300
C2—C3	1.384 (4)	C20—C21	1.403 (4)
C2—H2A	0.9300	C20—C26	1.457 (4)
C3—O5	1.384 (3)	C21—O12	1.380 (3)
C3—C4	1.397 (4)	C21—C22	1.382 (4)
C4—C5	1.405 (4)	C22—H22A	0.9300
C4—C7	1.456 (4)	C24—O11	1.212 (4)
C5—C6	1.384 (4)	C24—O12	1.382 (4)
C5—H5A	0.9300	C24—C25	1.442 (5)
C6—H6A	0.9300	C25—C26	1.345 (4)
C7—C8	1.347 (4)	C25—H25A	0.9300
C7—C11	1.510 (4)	C26—C27	1.509 (4)
C8—C9	1.434 (4)	C27—H27A	0.9600
C8—H8A	0.9300	C27—H27B	0.9600
C9—O6	1.220 (4)	C27—H27C	0.9600
C9—O5	1.392 (4)	C28—O8	1.462 (4)
C11—H11A	0.9600	C28—C30	1.529 (5)
C11—H11B	0.9600	C28—H28A	0.9700
C11—H11C	0.9600	C28—H28B	0.9700
C12—O3	1.472 (4)	C29—O7	1.465 (4)
C12—C14	1.521 (4)	C29—C30	1.528 (4)
C12—H12A	0.9700	C29—H29A	0.9700
C12—H12B	0.9700	C29—H29B	0.9700
C13—O4	1.467 (3)	C30—C32	1.527 (4)
C13—C14	1.514 (5)	C30—C31	1.534 (4)
C13—H13A	0.9700	C31—H31A	0.9600
C13—H13B	0.9700	C31—H31B	0.9600
C14—C15	1.535 (4)	C31—H31C	0.9600
C14—C16	1.540 (5)	C32—H32A	0.9600
C15—H15A	0.9600	C32—H32B	0.9600
C15—H15B	0.9600	C32—H32C	0.9600
			
O2—P1—O4	113.98 (14)	H16A—C16—H16C	109.5
O2—P1—O3	112.79 (15)	H16B—C16—H16C	109.5
O4—P1—O3	106.97 (12)	C22—C17—C18	121.8 (3)
O2—P1—O1	115.32 (14)	C22—C17—O10	115.7 (3)
O4—P1—O1	100.79 (12)	C18—C17—O10	122.5 (3)
O3—P1—O1	105.91 (13)	C19—C18—C17	118.6 (3)
O9—P2—O8	115.29 (14)	C19—C18—H18A	120.7
O9—P2—O7	114.16 (16)	C17—C18—H18A	120.7
O8—P2—O7	106.56 (13)	C18—C19—C20	121.7 (3)
O9—P2—O10	114.46 (14)	C18—C19—H19A	119.1
O8—P2—O10	100.43 (12)	C20—C19—H19A	119.1
O7—P2—O10	104.48 (12)	C19—C20—C21	116.9 (3)
C2—C1—C6	121.0 (3)	C19—C20—C26	124.5 (3)
C2—C1—O1	116.0 (3)	C21—C20—C26	118.5 (3)
C6—C1—O1	123.0 (3)	O12—C21—C22	115.9 (3)
C1—C2—C3	118.8 (3)	O12—C21—C20	121.4 (3)
C1—C2—H2A	120.6	C22—C21—C20	122.7 (3)
C3—C2—H2A	120.6	C17—C22—C21	118.2 (3)
O5—C3—C2	115.7 (3)	C17—C22—H22A	120.9
O5—C3—C4	121.6 (3)	C21—C22—H22A	120.9
C2—C3—C4	122.6 (3)	O11—C24—O12	116.3 (3)
C3—C4—C5	116.8 (3)	O11—C24—C25	126.7 (3)
C3—C4—C7	118.5 (3)	O12—C24—C25	117.0 (3)
C5—C4—C7	124.7 (3)	C26—C25—C24	123.8 (3)
C6—C5—C4	121.7 (3)	C26—C25—H25A	118.1
C6—C5—H5A	119.2	C24—C25—H25A	118.1
C4—C5—H5A	119.2	C25—C26—C20	117.9 (3)
C5—C6—C1	119.1 (3)	C25—C26—C27	121.8 (3)
C5—C6—H6A	120.4	C20—C26—C27	120.3 (3)
C1—C6—H6A	120.4	C26—C27—H27A	109.5
C8—C7—C4	118.2 (3)	C26—C27—H27B	109.5
C8—C7—C11	121.7 (3)	H27A—C27—H27B	109.5
C4—C7—C11	120.0 (3)	C26—C27—H27C	109.5
C7—C8—C9	123.4 (3)	H27A—C27—H27C	109.5
C7—C8—H8A	118.3	H27B—C27—H27C	109.5
C9—C8—H8A	118.3	O8—C28—C30	112.4 (3)
O6—C9—O5	115.8 (3)	O8—C28—H28A	109.1
O6—C9—C8	126.8 (3)	C30—C28—H28A	109.1
O5—C9—C8	117.5 (3)	O8—C28—H28B	109.1
C7—C11—H11A	109.5	C30—C28—H28B	109.1
C7—C11—H11B	109.5	H28A—C28—H28B	107.8
H11A—C11—H11B	109.5	O7—C29—C30	111.7 (2)
C7—C11—H11C	109.5	O7—C29—H29A	109.3
H11A—C11—H11C	109.5	C30—C29—H29A	109.3
H11B—C11—H11C	109.5	O7—C29—H29B	109.3
O3—C12—C14	111.6 (3)	C30—C29—H29B	109.3
O3—C12—H12A	109.3	H29A—C29—H29B	107.9
C14—C12—H12A	109.3	C32—C30—C29	110.8 (3)
O3—C12—H12B	109.3	C32—C30—C28	111.4 (3)
C14—C12—H12B	109.3	C29—C30—C28	107.9 (3)
H12A—C12—H12B	108.0	C32—C30—C31	111.0 (3)
O4—C13—C14	111.9 (3)	C29—C30—C31	107.8 (3)
O4—C13—H13A	109.2	C28—C30—C31	107.7 (3)
C14—C13—H13A	109.2	C30—C31—H31A	109.5
O4—C13—H13B	109.2	C30—C31—H31B	109.5
C14—C13—H13B	109.2	H31A—C31—H31B	109.5
H13A—C13—H13B	107.9	C30—C31—H31C	109.5
C13—C14—C12	108.5 (3)	H31A—C31—H31C	109.5
C13—C14—C15	108.1 (3)	H31B—C31—H31C	109.5
C12—C14—C15	108.3 (3)	C30—C32—H32A	109.5
C13—C14—C16	110.3 (3)	C30—C32—H32B	109.5
C12—C14—C16	110.4 (3)	H32A—C32—H32B	109.5
C15—C14—C16	111.1 (3)	C30—C32—H32C	109.5
C14—C15—H15A	109.5	H32A—C32—H32C	109.5
C14—C15—H15B	109.5	H32B—C32—H32C	109.5
H15A—C15—H15B	109.5	C1—O1—P1	126.35 (19)
C14—C15—H15C	109.5	C12—O3—P1	119.3 (2)
H15A—C15—H15C	109.5	C13—O4—P1	119.1 (2)
H15B—C15—H15C	109.5	C3—O5—C9	120.6 (3)
C14—C16—H16A	109.5	C29—O7—P2	117.2 (2)
C14—C16—H16B	109.5	C28—O8—P2	118.07 (19)
H16A—C16—H16B	109.5	C17—O10—P2	125.89 (19)
C14—C16—H16C	109.5	C21—O12—C24	121.4 (3)
			
C6—C1—C2—C3	1.3 (5)	C19—C20—C26—C25	−179.6 (3)
O1—C1—C2—C3	179.5 (3)	C21—C20—C26—C25	2.0 (5)
C1—C2—C3—O5	−179.3 (3)	C19—C20—C26—C27	0.5 (5)
C1—C2—C3—C4	0.9 (5)	C21—C20—C26—C27	−177.9 (3)
O5—C3—C4—C5	178.3 (3)	O7—C29—C30—C32	−63.5 (4)
C2—C3—C4—C5	−2.0 (5)	O7—C29—C30—C28	58.8 (4)
O5—C3—C4—C7	0.0 (5)	O7—C29—C30—C31	174.8 (3)
C2—C3—C4—C7	179.8 (3)	O8—C28—C30—C32	64.7 (4)
C3—C4—C5—C6	0.9 (5)	O8—C28—C30—C29	−57.2 (3)
C7—C4—C5—C6	179.0 (3)	O8—C28—C30—C31	−173.3 (3)
C4—C5—C6—C1	1.2 (5)	C2—C1—O1—P1	164.6 (2)
C2—C1—C6—C5	−2.4 (5)	C6—C1—O1—P1	−17.3 (5)
O1—C1—C6—C5	179.5 (3)	O2—P1—O1—C1	54.0 (3)
C3—C4—C7—C8	0.8 (5)	O4—P1—O1—C1	177.2 (3)
C5—C4—C7—C8	−177.3 (3)	O3—P1—O1—C1	−71.5 (3)
C3—C4—C7—C11	−178.1 (3)	C14—C12—O3—P1	−50.7 (3)
C5—C4—C7—C11	3.8 (5)	O2—P1—O3—C12	164.3 (2)
C4—C7—C8—C9	0.4 (5)	O4—P1—O3—C12	38.2 (2)
C11—C7—C8—C9	179.2 (3)	O1—P1—O3—C12	−68.7 (2)
C7—C8—C9—O6	177.8 (4)	C14—C13—O4—P1	52.3 (3)
C7—C8—C9—O5	−2.3 (5)	O2—P1—O4—C13	−164.1 (2)
O4—C13—C14—C12	−58.6 (4)	O3—P1—O4—C13	−38.8 (2)
O4—C13—C14—C15	−175.9 (3)	O1—P1—O4—C13	71.7 (2)
O4—C13—C14—C16	62.5 (3)	C2—C3—O5—C9	178.2 (3)
O3—C12—C14—C13	57.7 (4)	C4—C3—O5—C9	−2.1 (5)
O3—C12—C14—C15	174.9 (3)	O6—C9—O5—C3	−177.0 (3)
O3—C12—C14—C16	−63.3 (4)	C8—C9—O5—C3	3.1 (5)
C22—C17—C18—C19	−1.0 (5)	C30—C29—O7—P2	−55.6 (3)
O10—C17—C18—C19	−179.6 (3)	O9—P2—O7—C29	172.4 (2)
C17—C18—C19—C20	1.7 (5)	O8—P2—O7—C29	43.9 (2)
C18—C19—C20—C21	−1.2 (5)	O10—P2—O7—C29	−61.9 (2)
C18—C19—C20—C26	−179.6 (3)	C30—C28—O8—P2	52.4 (3)
C19—C20—C21—O12	179.2 (3)	O9—P2—O8—C28	−170.1 (2)
C26—C20—C21—O12	−2.3 (5)	O7—P2—O8—C28	−42.3 (3)
C19—C20—C21—C22	0.0 (5)	O10—P2—O8—C28	66.3 (2)
C26—C20—C21—C22	178.5 (3)	C22—C17—O10—P2	168.6 (2)
C18—C17—C22—C21	−0.1 (5)	C18—C17—O10—P2	−12.7 (4)
O10—C17—C22—C21	178.7 (3)	O9—P2—O10—C17	66.6 (3)
O12—C21—C22—C17	−178.7 (3)	O8—P2—O10—C17	−169.3 (2)
C20—C21—C22—C17	0.6 (5)	O7—P2—O10—C17	−59.0 (3)
O11—C24—C25—C26	176.9 (4)	C22—C21—O12—C24	179.6 (3)
O12—C24—C25—C26	−1.9 (5)	C20—C21—O12—C24	0.3 (5)
C24—C25—C26—C20	0.1 (5)	O11—C24—O12—C21	−177.2 (3)
C24—C25—C26—C27	180.0 (3)	C25—C24—O12—C21	1.7 (5)

### Hydrogen-bond geometry (Å, º)

**Table:**

*D*—H···*A*	*D*—H	H···*A*	*D*···*A*	*D*—H···*A*
C8—H8*A*···O2^i^	0.93	2.36	3.223 (5)	155
C13—H13*B*···O11^ii^	0.97	2.48	3.250 (5)	136

Symmetry codes: (i) −*x*, *y*+1/2, −*z*+3/2; (ii) −*x*+1, −*y*+1, −*z*+1.

## References

[bb1] Babu, B. H., Prasad, G. S., Reddy, C. S. & Raju, C. N. (2008). *Heteroatom* *Chem.* **19**, 256–260.

[bb2] Bruker (2007). *APEX2*, *SAINT* and *SADABS* Bruker AXS Inc., Madison, Wisconsin, USA.

[bb3] Li, X., Ou, Y.-X. & Shi, Y. (2002). *Polym. Degrad. Stab.* **77**, 383–390.

[bb4] Li, Z.-Q., Sheng, X.-J., Zuo, N., Ren, Q.-Y. & He, H.-W. (2006). *Acta Cryst.* E**62**, o3501–o3502.

[bb5] Raghu, K. V. & Reddy, C. D. (1996). *Indian J. Chem. Sect. B*, **35**, 1228–1232.

[bb6] Sheldrick, G. M. (2008). *Acta Cryst.* A**64**, 112–122. 10.1107/S0108767307043930 18156677

[bb7] Sierosławski, K., Ślepokura, K. & Lis, T. (2006). *Acta Cryst.* E**62**, m560–m562. 10.1107/S0108270106017811 16823215

[bb8] Zhou, X., Wang, X.-B. & Kong, L.-Y. (2006). *Acta Cryst.* C**62**, o58–o61. 10.1107/S0108270105040928 16456285

